# Multiple Plant Growth-Promotion Traits in Endophytic Bacteria Retrieved in the Vegetative Stage From Passionflower

**DOI:** 10.3389/fpls.2020.621740

**Published:** 2021-01-18

**Authors:** Luis Gabriel Cueva-Yesquén, Marcela Cristina Goulart, Derlene Attili de Angelis, Marcos Nopper Alves, Fabiana Fantinatti-Garboggini

**Affiliations:** ^1^Graduate Program in Genetics and Molecular Biology, Institute of Biology, University of Campinas, Campinas, Brazil; ^2^Division of Microbial Resources, Research Center for Chemistry, Biology and Agriculture (CPQBA), University of Campinas, Paulínia, Brazil; ^3^Division of Agrotechnology, Research Center for Chemistry, Biology and Agriculture (CPQBA), University of Campinas, Paulínia, Brazil

**Keywords:** Passiflora incarnata, inoculant, Cape gooseberry, PGP bacteria, PGP genes

## Abstract

Bacteria exhibiting beneficial traits like increasing the bioavailability of essential nutrients and modulating hormone levels in plants are known as plant growth promoting (PGP) bacteria. The occurrence of this specific group of bacteria in the endophytic environment may reflect the decisive role they play in a particular condition. This study aimed to determine the taxonomical diversity of the culturable bacterial endophytes, isolated in the vegetative stage of passionflower (*Passiflora incarnata*), and assess its potential to promote plant growth by phenotypic and genotypic approaches. The sequencing and phylogenetic analysis of the 16S rRNA gene allowed us to classify 58 bacterial endophytes into nine genera. *Bacillus* (70.7%) was the most dominant genus, followed by *Pseudomonas* (8.6%) and *Pantoea* (6.9%). A few isolates belonged to *Rhodococcus* and *Paenibacillus*, whereas the genera *Lysinibacillus*, *Microvirga*, *Xanthomonas*, and *Leclercia* were represented by only one isolate. The strains were tested for nitrogen fixation, phosphate solubilization, indole-acetic-acid synthesis, and siderophore production. Moreover, PGP related genes (*nifH*, *ipdC*, *asb*, and *AcPho*) were detected by PCR-based screening. Most of the isolates (94.8%) displayed a potential for at least one of the PGP traits tested by biochemical assays or PCR-based screening. Nine strains were selected based on results from both approaches and were evaluated for boosting the Cape gooseberry (*Physalis peruviana*) germination and growth. All tested isolates improved germination *in vitro*, and the majority (78%) increased growth parameters *in vivo*. The results suggested that most of culturable bacteria inhabiting *P. incarnata* in the vegetative stage could be used as probiotics for agricultural systems. Besides, their occurrence may be associated with specific physiological needs typical of this development stage.

## Introduction

Endophytes can be defined as microorganisms living inside the plant tissues without causing any apparent disease (Compant et al., 2010; Hardoim et al., 2015). They are mainly located in the extracellular fluids and, in some cases, inside the cells (Compant et al., 2010), where they may interact with each other and with the host to assemble a specific community in distinct compartments of the plant. The structure and composition of endophytic communities are determined by environment and plant-associated factors, such as the plant genotype, the developmental stage, phenology, and edaphic properties (Seghers et al., 2004; Van Overbeek and Van Elsas et al., 2008; de Silva et al., 2016; Goulart et al., 2019).

Endophytes are widely known for maintaining and boosting the plant health and development (Santoyo et al., 2016), while plants provide a complex niche constituted by specific abiotic and biotic factors supporting the endophytic colonization (McCully, 2001). However, due to different nutritional needs in different developmental stages, the physiology of plants varies across the life cycle. In the vegetative stage, the demand for essential nutrients, such as nitrogen, phosphorus, and iron, are often increased; however, these nutrients are poorly supplied or unavailable for plant uptake (López-Arredondo et al., 2013). Moreover, the role of indole-3-acetic acid (IAA) is so fundamental for vegetative growth that plants exhibit a higher capacity to synthesize this phytohormone during the vegetative stage (Ljung et al., 2001). Endophytic bacteria have been widely associated to mobilization of essential nutrients and synthesis of plant growth regulators (Santoyo et al., 2016). For example, various endophytic bacterial strains have shown beneficial traits, including nitrogen fixation, inorganic phosphorus solubilization, siderophores secretion, and IAA synthesis (Crowley, 2006; Gupta et al., 2012; Sharma et al., 2013; Glick, 2014). This specific group of bacteria is commonly known as plant growth promoting (PGP) bacteria. In general, it is thought that PGP bacteria can positively affect soil fertility and nutrient uptake in plants (Rashid et al., 2016; Bargaz et al., 2018; Kumar et al., 2020). These characteristics include them into plant probiotics, which promote the biological process directly related to plant development and protection (Bharti et al., 2017). The beneficial effect of probiotics on plants is reflected in the improvement of production and nutritional quality and the recovery of natural equilibrium in agro-ecosystems (Woo and Pepe, 2018).

Passionflower (*Passiflora incarnata*) is a tropical plant widely used as traditional herbal medicine. The phytochemical composition of passionflower includes mainly alkaloids and flavonoids, which support their therapeutic use to treat anxiety, nervousness, constipation, dyspepsia, and insomnia (Dhawan et al., 2001). These pharmacological properties allowed it to be included in the national pharmacopeias of France, Germany, and Switzerland. In addition, several *P. incarnata* derivative preparations have been manufactured and delivered as medicinal products and food supplements around the world (Miroddi et al., 2013). This plant has tendril-climbing stems and three-lobed leaves in its vegetative stage from December to January, and it blooms with showy and fragrant flowers from April to November (Fuentes et al., 2000). Passionflower occurs in sandy and well-drained soils, woods with low moisture and open areas (Miroddi et al., 2013). It is considered a “heavy feeder” plant since it needs a balanced fertilizer that supplies the macronutrients and micronutrients, which are often present in unavailable forms in the soil and have a critical role in its vegetative growth. Nevertheless, the pharmaceutical industry restricts the use of chemical fertilizer and pesticides in its culture, since they can compromise human food security (Björnberg et al., 2015). These conditions create a challenger scenario for passionflower culture.

The present knowledge of plant microbiome suggests that, when a plant host faces unfavorable conditions, it alters its physiological structure and consequently the plant-microbe and microbe-microbe interactions (Uroz et al., 2019). These changes can stimulate the recruitment and increase of beneficial microbes to meet plant physiological needs (Liu et al., 2020). Thus, the occurrence of microorganisms with traits related to essential nutrients acquisition and synthesis of growth regulators might suggest their role in the passionflower culture. We hypothesize that because of the environmental constraints and physiological needs in which *P. incarnata* is found, its associated microbiota contributes with beneficial functions for plant development. This study aims at determining the diversity of culturable endophytic bacteria retrieved from *P. incarnata* in the vegetative stage and at assessing their plant growth promotion traits.

## Materials and Methods

### Bacterial Isolates From *Passiflora incarnata*

Fifty-eight endophytic bacteria, provided by the Microbial Resources Division of the Research Center for Chemistry, Biology and Agriculture (CPQBA), University of Campinas, were characterized in this study. These bacteria were isolated from leaf tissues in the vegetative stage of *P. incarnata* by Goulart et al. (2019). The passionflower leaves were collected in January 2015 from the Centroflora Group agricultural fields located at Botucatu, São Paulo, Brazil. The culture of *P. incarnata* in these fields is exempt from the application of any chemical fertilizers. Leaves were surface-sterilized and aseptically grounded in Phosphate Buffer Saline (PBS). Then, the suspensions were serially diluted to10^−4^. Aliquots (100 μl) of each 10-fold dilution were plated in seven culture media including M9 minimal medium, Gause’s synthetic agar, Tap Water Yeast Extract agar, Humic acid-Vitamin agar, Glycerol-asparagine agar, Chitin medium (Zhao et al., 2012), and 869 medium (Eevers et al., 2015). For this study, all isolates were sub-cultured in Trypticase Soy Agar (TSA) at 28°C for 48–96 h.

### 16S rRNA Gene Sequencing and Phylogenetic Analysis

The genomic DNA of bacteria was extracted according to a modified protocol of Van Soolingen et al. (1993). The 16S rRNA gene was partially amplified by PCR using the universal bacterial primers 10F (5'-AGAGTTTGATCCTGGCTCAG-3') and 1501R (5'-AAGGAGGTGATCCAGCCGCA-3'; Lane, 1991). The PCR reaction was performed in 25 μl final volume containing dNTPs (0.2 mM each), 1X reaction buffer (20 mM Tris, pH 8.4), 1.5 mM MgCl_2_, 0.5 μM each primer, 1 U of Taq DNA polymerase, and 10 ng of template DNA. The PCR cycling protocol consisted of an initial denaturation at 94°C for 4 min, followed by 32 cycles of 94°C for 1 min, 55°C for 1 min, and 72°C for 3 min, and a final extension at 72°C for 5 min. The PCR amplified products were run on a 1% (v/w) agarose gel stained with SYBR™ Safe (Thermo Fisher Scientific) and purified using the GFX™ PCR DNA Purification kit (GE Healthcare Life Sciences, Germany). Amplicons were sequenced by the Sanger method with BigDye Terminator v3.1 Cycle Sequencing Kit (Applied Biosystems Life Technologies) using the same primers of amplification and the internal primers 765F (5'-ATTAGATACCCTGGTAG-3') and 785R (5'-ACCAGGGTATCTAATCCTGT-3'). The sequencing cycling protocol consisted of an initial denaturation at 96°C for 1 min, followed by 30 cycles of 96°C for 15 s, 50°C for 15 s, and 60°C for 4 min. The reaction products were sequenced on an ABI3500XL Series (Applied Biosystems) sequencer. The sequences were assembled in contigs using BioEdit 7.2.6.1 software (Hall, 1999) and compared with the reference 16S rRNA gene sequences available in the EzBioCloud platform^1^ (Yoon et al., 2017). Newly generated sequences were deposited in GenBank under accession numbers MG778707 to MG778907. The phylogenetically closest sequences were selected and used for subsequent phylogenetic analyses. The 16S rDNA sequences of the isolates and reference bacterial sequences were aligned using CLUSTAL W (Thompson et al., 1994), and the substitution model was determined with MODELTEST from MEGA X software (Tamura et al., 2013). The clustering was performed using the Neighbor-Joining algorithm, and evolutionary distances were computed with the Kimura two-parameter model. The support of nodes was estimated by bootstrapping with 1,000 replications (Felsenstein, 1985). The phylogenetic analysis resulting from MEGA X was exported in the Newick format to create a circular cladogram in iTOL^2^ (Letunic and Bork, 2016).

### Biochemical Assays for PGP Traits

#### Growth on N-Free Medium

The endophytic isolates were tested for their ability to fix or scavenge N using a nitrogen-free medium. Bacterial cultures were grown overnight at 30°C in Trypticase Soy Broth (TSB) medium, washed twice, and resuspended in PBS (pH 7.4). The bacterial concentration was adjusted for OD_600_ 0.5. A 30 μl aliquot of each bacterial suspension was inoculated into 10 ml vials containing 4 ml of semi-solid New Fabian broth (NFb) medium (Baldani et al., 1986) and incubated at 28°C. The bacterial growth was confirmed from 72 h incubation by forming a sub-surface pellicle on the culture medium. The diazotrophic potential was demonstrated through successive re-inoculations in NFb medium. The experiments were conducted in triplicate.

#### Phosphate Solubilization

The ability of endophytic bacteria to solubilize inorganic phosphorous was evaluated according to Mehta and Nautiyal (2001). All bacterial isolates were first grown overnight at 30°C in TSB medium to obtain OD600 0.5. A 10 μl aliquot of each bacterial culture was inoculated in Petri dishes containing the solid National Botanical Research Institute’s Phosphate (NBRIP) medium. The plates were incubated at 30°C for 15 days. The development of a transparent halo zone around the colony revealed the phosphate-solubilizing ability of the isolate. To estimate the phosphate-solubilizing ability quantitatively, the Solubilization Index (SI) was calculated as follows: SI = A/B, where A is the colony diameter + halo zone diameter, and B is the colony diameter (Edi-Premono, 1996).The isolates were grouped according to Silva Filho and Vidor (2000), in bacteria with low (SI < 2), intermediate (2 < SI < 3), and high (SI > 3) solubilization potential. The experiments were conducted in triplicate.

#### IAA-Like Compounds Production

Indole-3-acetic acid production was estimated by growing the isolates on a TSB medium containing 5 mM L-tryptophan, at 30°C in a rotary shaker at 150 rpm for 48 h in the dark. Bacterial cultures were centrifuged at 8,000 rpm for 15 min. An aliquot (1 ml) of supernatant was mixed with 2 ml of Salkowski reagent (0.5 M FeCl_3_.6H_2_O in 35% HClO_4_) and incubated in the dark for 30 min at room temperature (Tang and Bonner, 1948). The UV-Vis absorption spectra were measured spectrophotometrically at 530 nm. A standard curve with known concentrations (0.5–120 μg/ml) of IAA (Sigma-Aldrich) was used to determine the amount of IAA produced. The experiments were conducted in triplicate.

#### Siderophore Production

Siderophore production was determined qualitatively on Chrome Azurol S (CAS) supplemented Blue Agar plates (Schwyn and Neilands, 1987). The bacterial isolates were first grown overnight at 30°C in TSB medium to obtain OD_600_ 0.5. A 10 μl aliquot of each bacterial culture was inoculated onto a diffusion disc placed on the CAS-Blue Agar (Hussein and Joo, 2014). The diffusion disc method was used to avoid the toxic effect of Hexadecyltrimethylammonium (HDTMA; Chimwamurombe et al., 2016), responsible for the blue color of the medium. Plates were incubated for 72 h at 30°C and observed daily until a yellow orange halo was seen around the colony. The experiments were conducted in triplicate.

### Detection of PGP Related Genes

A PCR based approach was applied to confirm and complement the information provided by biochemical assays or even to reveal new PGP potentials of bacteria. The ability to reduce atmospheric nitrogen was evaluated by amplifying the gene encoding for nitrogenase reductase *nifH*. For this purpose, a nested PCR protocol was performed using the primers *nifH* (forA) and *nifH* (reverse) for a first reaction and the primers *nifH* (forB) and *nifH* (reverse) in the second reaction (Table 1; Zehr and McReynolds, 1989). The PCR conditions were as indicated by Burgmann et al. (2004). Briefly, the first amplification was performed in a final volume of 25 μl, containing 10 ng of genomic DNA, 2 μM of each primer, 0.2 mM of each dNTP, 2 mM MgCl_2_, 1 U of Taq Polymerase Recombinant (Invitrogen), and 1x of PCR buffer. The nested reaction was carried out with 1 μl of the PCR product added to a new mixture prepared as before. The annealing conditions were 30 s at 55° C and 30 s at 53° C for the first and second reactions, respectively. As a positive control, the gDNA of *Gluconacetobacter diazotrophicus* PAl 5, known for its nitrogen fixation activity, was used. The PCR products were separated by electrophoresis in a 1% (v/w) agarose gel stained with SYBR™ Safe (Thermo Fisher Scientific). The fragments with the expected size were sequenced and analyzed by BLASTX using “non-redundant” protein sequences database from NCBI. The matches with identity >80% were considered.

**Table 1:** Primers used to amplify genes associated with plant growth promotion.

Gene	Size of gene (pb)	Primer sequence (5'→3')	Amplicon (pb)	Reference
*nifH*	896	*forA*-GCIWTITAYGGNAARGGNGG	371	Zehr and Reynolds, 1989
		*forB*-GGITGTGAYCCNAAVGCNGA		
		*rever*-GCRTAIABNGCCATCATYTC		
*ipdC*	1,809	CAYTTGAAAACKCAMTATACTG	1,715	Raddadi et al. (2008)
		AAGAATTTGYWKGCCGAATCT		
*asb*	1,685	GAGAATGGATTACAGAGGAT	1,685	Raddadi et al. (2008)
		TTATGAACGAACAGCCACTT		
*AcPho*	828	AAGAGGGGCATTACCACTTTATTA	734	Raddadi et al. (2008)
		CGCCTTCCCAATCRCCATACAT		

Indole-3-acetic acid production was screened by partial amplification of the *ipdC*, the gene encoding for indole-3-pyruvate (IPA) decarboxylase, the most important enzyme in the indole-3-pyruvic acid (IPyA) pathway. The IPyA is the pathway used by most beneficial bacteria (*Azospirillum*, *Bacillus*, *Bradyrhizobium*, *Enterobacter cloacae*, *Paenibacillus*, *Pseudomonas*, and *Rhizobium*; Spaepen and Vanderleyden, 2011). The ability to synthesize siderophores was assessed by partially amplifying the *asb* gene that encodes for petrobactin, a catechol-type siderophore commonly secreted by *Bacillus* spp. (Koppisch et al., 2008). To evaluate the potential of solubilizing phosphates, we amplified gene encoding for the acid phosphatase, an enzyme involved in the mineralization of most organic phosphorus compounds from soil (El-Sawah et al., 1993). PCR amplifications of *ipdC*, *asb*, and *AcPho* were conducted in all isolates using gene specific primers (Table 1) as described by Raddadi et al. (2008). The PCR reaction was performed in a final volume of 25 μl containing dNTPs (0.2 mM each), 1x reaction buffer (20 mM Tris, pH 8.4), 2.5 mM MgCl_2_, 1.0 μM of each primer, 1 U of Taq DNA polymerase, and 50 ng of template DNA. The PCR cycling protocol consisted of an initial denaturation at 94°C for 2 min, followed by 30 cycles of 94°C for 1 min, 55°C (*asb* and *AcPho*) and 50°C (*ipdC*) for 45 s and 72°C for 2 min, followed by a final extension at 72°C for 5 min. The PCR amplified products were analyzed by 1% (v/w) agarose gel electrophoresis. The fragments with the correct size were sequenced and analyzed by BLASTX. The isolates with one or more PGP traits (by phenotypic and genotypic approaches) were intersected in an UpSet graphic using the Intervene platform (Khan and Mathelier, 2017).

### Evaluation of Plant Growth Promotion *in vivo*

#### Effect on the Cape Gooseberry (*Physalis peruviana*) Germination

Cape gooseberry is a plant of economic importance which has gained recognition in the international market due to its nutritional value and versatility to be consumed. Seeds are the main propagation method in the Cape gooseberry culture, due to the high seed number per fruit (Puente et al., 2011). Based on the biochemical assays, nine bacterial isolates with multiple PGP traits (three or more) were selected and used to evaluate their effect on Cape gooseberry seedling vigor and germination. Seeds of *Physalis peruviana* were surface sterilized using a 3% sodium hypochlorite solution for 10 min, then washed five times with sterile distilled water for 3 min each. This plant genotype was obtained from the Collection of Medicinal Plants, at the Research Center for Chemistry, Biology and Agriculture (CPQBA), Brazil. The inocula were prepared by growing the selected isolates on TSB at 28°C for 20 h with shaking (150 rpm). Bacterial cells were harvested by centrifugation at 9,000 rpm for 10 min at 4°C, and each pellet was washed three times with the PBS solution. The pellets were suspended in the PBS solution and adjusted to 0.5 OD_590_. Surface-sterilized seeds were dipped into bacterial inocula for 60 min and dried in a laminar flow bench at room temperature. Fifty seeds inoculated with each endophytic isolate were spread on two layers of moistened filter paper on the Petri plates. For the control treatment, 50 surface-sterilized seeds treated with sterilized PBS were also established. Inoculated and control plates were incubated in a light incubator (16 h in a day) at 28 ± 2°C for 10 days. To maintain sufficient moisture for germination, 1 ml of sterilized distilled water was added every 24 h. Germination was considered to occur once the radicles reached half of the seed length. The root and shoot length were measured after 10 days. The germination speed index (GSI) was calculated according to Maguire (1962) and sprouted seeds were counted 6, 8, and 10 days after test initiation. The experiment was carried out with three replicates.

Germination (%) = number of seeds germinated/ total number of seeds × 100

Vigor index = % germination × total plant length (mm)

#### Effect of Endophytic Bacteria on Cape Gooseberry Growth

The nine selected isolates were used to determine the growth promoting capability in Cape gooseberry plants. Surface-sterilized seeds were inoculated as described above with selected isolates. A set of seeds were treated with PBS (control treatment). Treated seeds were sown 1 cm deep in the commercial substrate (Tropstrato Hortaliças Mix, Brazil) contained in 108-plug trays. The substrate was autoclaved twice at 24 h intervals at 121°C and 15 psi for 30 min. After 15 days, germinated embryos were subjected to two additional inoculations. Bacterial suspensions, prepared according to “Effect on the Cape Gooseberry (*Physalis peruviana*) Germination” section, were applied to the plant base at 2 and 7 days after germination. Seedlings with similar growth status were selected from each treatment for further analysis. Plants were grown for 8 weeks in a net house, and seven plants (replicates) from each treatment were harvested for measuring the dry matter, root and shoot lengths, a and b chlorophyll, and macronutrients and micronutrients.

### Statistical Analysis

A completely randomized design was used for pot experiments, with seven replications for each treatment. Arithmetic means and standard deviations were calculated. Significant differences were assessed by one-way analysis of variance (one-way ANOVA), *post-hoc* test Tukey HSD. ANOVA assumptions were revised by the equal variance test (Levene Median) and normality test (Kolmogorov-Smirnov and Lilliefors tests). All statistical analyses were performed in Sigma 12.0.

## Results

### Phylogenetic Analysis of Bacterial Endophytic Isolates

**Figure 1:**
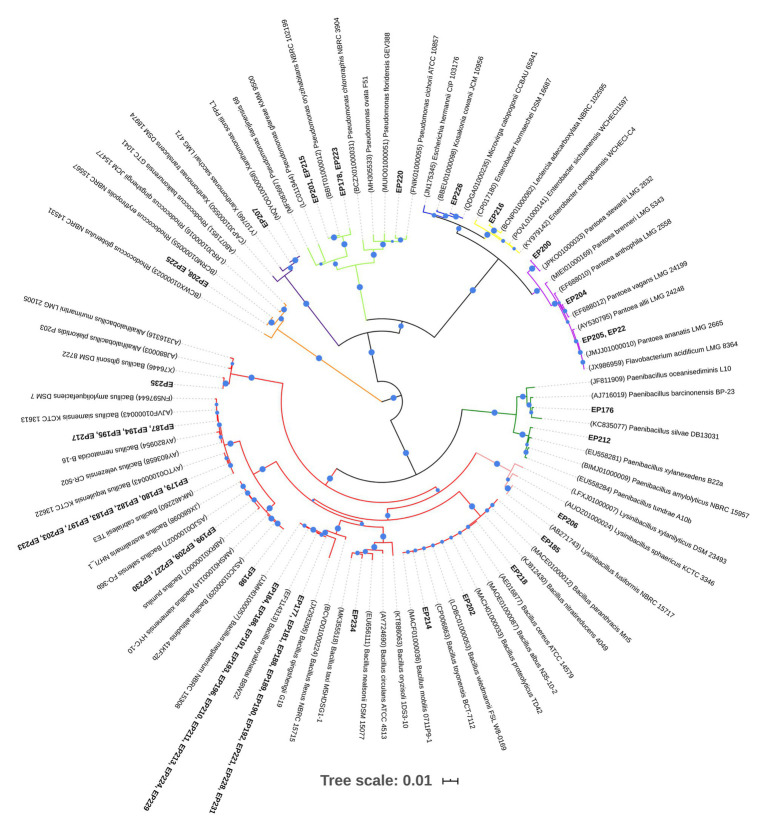
Neighbor-joining phylogenetic tree based on partial 16S rRNA sequences of endophytic bacteria isolated from *Passiflora incarnata* leaves and reference sequences from EzBioCloud. The branch colors indicate different bacterial genera. Only bootstrap values equal and greater than 60% are displayed as circles with increasing size up to 100%. Accession numbers from references sequences are in parentheses.

The partial 16S rRNA gene sequencing from 58 bacteria provided sequences of sufficient length (mean length of 1,290 bp) to carry out the phylogenetic analysis. The sequences were submitted to the identify server of the EzBioCloud platform to recover the closest reference sequences. All sequences showed >99–100% similarity with reference sequences (Supplementary Table S1). The multiple sequence alignment in ClustalW generated 1,252 positions and it was used for constructing the phylogenetic tree (Figure 1), which showed well-supported clades and allowed to us determine the taxonomic affiliations of all isolates. However, sequences of EP178 and EP223 isolates did not group with any reference sequence. In a further phylogenetic analysis (Supplementary Figure S1), these sequences also formed a separated taxon supported by a high bootstrap value (100).

The phylogenetic analysis from 58 isolates allowed us to classify them into three phyla: Firmicutes, Proteobacteria, and Actinobacteria. The majority of the isolates (41/58) belong to the Firmicutes phylum, represented by Bacillaceae and Paenibacillaceae families. Proteobacteria was the second largest phylum (15/58) dominated by Enterobacteriaceae, Pseudomonadaceae, and Xanthomonadaceae. Bacteria belonging to the Actinobacteria phylum (2/58) were uniquely related to the Nocardiaceae family. The taxonomic affiliations of isolates, at the genera level, revealed that *Bacillus* (70.7%) was the dominant bacterial genus. Based on the tree topology, *Bacillus* sequences (marked in red color in Figure 1) formed six clades, one of them included sequences from 19 isolates (closely related to *Bacillus megaterium* and *Bacillus aryabhattai*). The EP206 isolate sequence clustered in the *Lysinibacillus* genus monophyletic group (marked in pink color). The *Paenibacillus* cluster (marked in green color) contained only two endophytic isolates (EP176 and EP212), which were distributed in two different clades. The second most abundant genus was *Pseudomonas* (8.6%), which was comprised of two clades (marked in light green), and their isolates were taxonomically associated to the species, *Pseudomonas cichorii* (EP220) and *Pseudomonas oryzihabitans* (EP201 and EP215), while the sequences of EP178 and EP223 grouped with *Pseudomonas* spp. Sequences from the *Pantoea* genus (marked in lilac color) formed three clades holding four endophytic isolates (EP200, EP204, EP205, and EP222). The others isolate belonging to Proteobacteria were distributed in the *Microvirga*, *Xanthomonas*, and *Leclercia* genera. The Actinobacteria strains (EP225 and EP208) were uniquely associated with the *Rhodococcus* genus and represented 3.4% of total bacterial endophytes.

### Detection of PGP Traits

**Figure 2:**
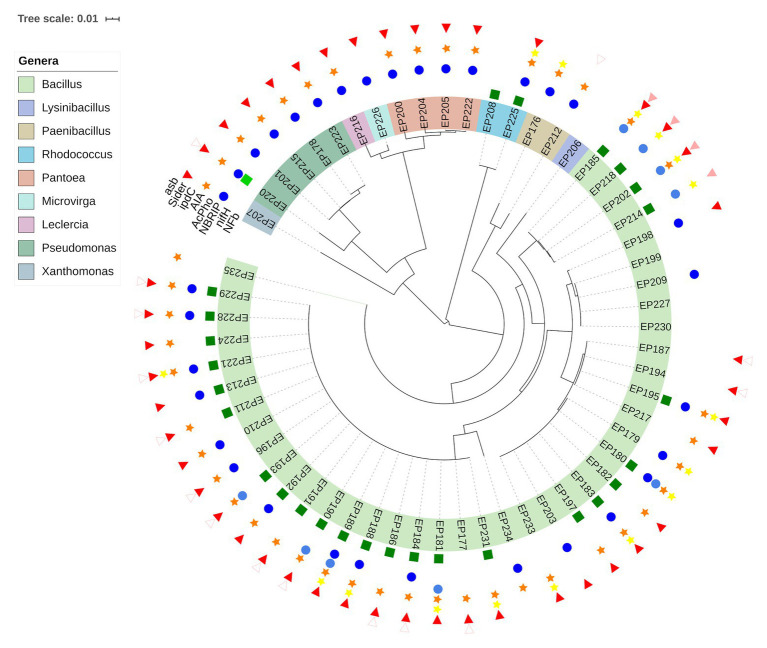
Diversity of PGP traits among endophytic bacteria according to their taxonomic affiliations inferred from phylogenetic analysis of 16S rRNA gene sequences. Growth on a N-free media (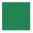); detection of the *nifH* gene(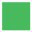); formation of halo on a NBRIP medium (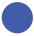);detection of the *AcPho* gene (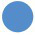); IAA-like compound production (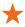); detection of the *ipdC* gene (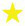); formation of orange halo on a CAS Agar (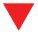); detection of the *asb* gene (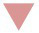); and unspecific amplification of a gene involved iron metabolism (*tatA*; 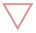).

The ability of endophytic bacteria to improve plant growth was characterized by a phenotypic approach. The biochemical assays were addressed to reveal the potential of strains to favor essential nutrient acquisition (nitrogen, phosphates and iron) and to synthesize a plant growth regulator (indol acetic acid). The results obtained are presented in Figure 2 (Supplementary Table S2). The assay on the semi-solid NFb medium allowed us to identify diazotrophic/N-scavenging strains. Twenty-six endophytic strains were able to grow in the N-free medium, forming a sub-surface pellicle, even after two successive inoculations. Most of the positive strains (92%) were related to the *Bacillus* genus, and only two (EP208 and EP225) belonged to *Rhodococcus*.

Thirty-three strains (56.9%) showed the ability to solubilize inorganic phosphates, Ca_3_(PO_4_)_2_, on the solid NBRIP medium. The bacteria that formed a halo around the colony were considered positive for phosphate solubilization. From the positive strains, 18 were affiliated to the *Bacillus* genus, while *Pseudomonas* and *Pantoea* were represented by five and four, respectively. The SI was calculated for the halo-forming isolates and is shown in Supplementary Table S2. Based on this index, the best strain in solubilizing phosphate was EP223, which was assigned as *Pseudomonas* spp. Furthermore, *Pseudomonas* was the genus with the highest number of strains with high solubilization potential. At the same time, most of the positive strains were placed in the intermediate potential group and largely associated with the *Bacillus* genus (Supplementary Figure S2).

Regarding IAA production, 44 isolates (75.8%) were able to synthesize IAA-like molecules when grown in a liquid medium supplemented with tryptophan. The IAA concentrations detected varied from 1.01 to 6.04 μg/ml. The values calculated for all isolates are shown in Supplementary Table S2. All strains belonging to *Pseudomonas*, *Pantoea* and *Paenibacillus* produced IAA-like compounds, but *Bacillus* was the genus with the highest number of positive strains. *Xanthomonas*, *Leclercia*, and *Rhodococcus* had one each positive strain for this test. The two *Paenibacillus* strains produced a mean of 5.35 μg/ml, the highest value among all genera. Nevertheless, EP229 (associated with *Bacillus*) was the strain that exhibited the highest IAA value (6.04 μg/ml).

Siderophore production was screened by using the CAS agar medium. Most of the endophytic strains (77.6%) formed orange halos around the bacterial colony, indicating that chelating agents capable of capturing the iron were secreted. Positive strains were mostly associated with *Bacillus*, followed by *Pseudomonas* (5) and *Pantoea* (4) strains. Members of *Xanthomonas*, *Leclercia*, *Rhodococcus*, and *Microvirga* were represented by only one positive strain.

### Screening of PGP Traits by PCR

Endophytic bacteria were evaluated by harboring genes related to plant growth promotion using a genotypic approach. The results obtained from this approach are presented in Figure 2 (Supplementary Table S2). The amplification of the *nifH* gene was performed to confirm the diazotrophic potential. A fragment (~371 bp) from the *nifH* gene was amplified only in the EP220 strain, which was closely related to *P. cichorii*. The BLASTX analysis showed that the deduced sequence from this strain shared 86% identity with a nitrogenase iron protein from *Insolitispirillum peregrinum*. This result is the first report of the presence of the *nifH* gene from a *P. cichorii* strain.

The *AcPho* gene was detected in nine strains. Conserved domains related to acid phosphatase enzyme were detected from amplified sequences. Strains carrying *AcPho* sequences were exclusively associated with the *Bacillus* genus, likely because the primer design was addressed for *Bacillus thuringiensis* strains (Raddadi et al., 2008).

Partial amplification of the *ipdC* gene was a success in 16 endophytic strains. Conserved domains related to the IPA decarboxylase enzyme were detected by the BLASTX analysis. Most strains carrying *ipdC* sequences were associated with the *Bacillus* genus. The *Rhodococcus* and *Paenibacillus* genera had only one representative in the genotypic approach.

However, *asb* gene was partially amplified in four strains (EP185, EP202, EP214, and EP218), which were associated with *Bacillus*. The BLASTX analysis detected conserved domains related to siderophore synthesis proteins, such as Aerobactin. Interestingly, an unspecific fragment of approximately 1,000 bp (Supplementary Figure S3) was amplified in 15 strains: 13 belonging to *Bacillus* and the other two to *Paenibacillus* and *Pseudomonas*. The sequencing and analysis of this unspecific fragment determined that it possessed conserved domains related to the TatA/TatE subunit of the translocase A protein, which transports proteins across bacterial cytoplasmatic membrane.

### The Dominant Groups Exhibited PGP Traits

**Figure 3:**
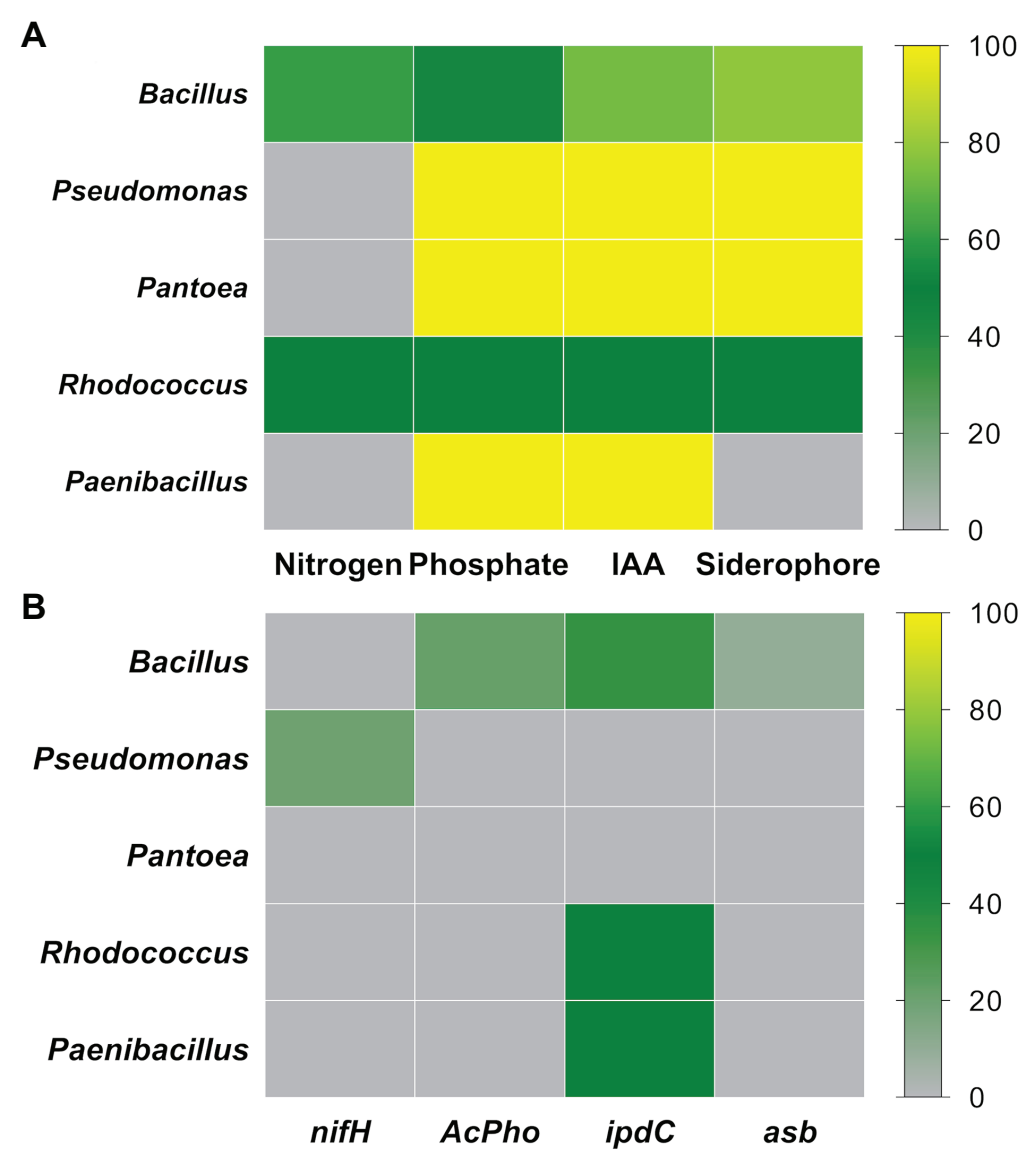
Relative abundance of PGP strains in the five most dominant genera: **(A)** PGP activities tested by biochemical assays and **(B)** PGP activities detected by PCR-based approach.

The strains belonging to the genera *Bacillus*, *Pseudomonas*, *Pantoea*, *Rhodococcus*, and *Paenibacillus* account to ~93% of the total endophytic bacteria retrieved from *P. incarnata* leaves in the vegetative stage. The plant growth-promoting trait abundance was evaluated on the five most abundant members (genera) mentioned before. Based on the biochemical tests (Figure 3A), the phosphates solubilizing ability varied from 44% in *Bacillus* and 55% in *Rhodococcus* to 100% in *Pseudomonas*, *Pantoea*, and *Paenibacillus* strains, while IAA-like compounds were detected in 50% of *Rhodococcus*, 73.2% of *Bacillus*, and 100% of *Pseudomonas*, *Pantoea*, and *Paenibacillus* strains. Siderophore production was exhibited in 100% of *Pseudomonas* and *Pantoea*, 78% of *Bacillus*, and 50% of *Rhodococcus* strains, while only 50% of *Rhodococcus* and 60% of *Bacillus* strains have grown forming a sub-surface pellicle in the NFb medium. Regarding the PCR-based approach (Figure 3B), the *nifH* gene was only detected in one out of *Pseudomonas* strains (EP220). None of the other genera had representatives carrying this gene. Only in the *Bacillus* strains, the *asb* and *AcPho* sequences were amplified. Unlikely, the *ipdC* gene was more frequently encountered among tested strains since it was detected in 34.1% of *Bacillus*, 50% of *Rhodococcus*, and 50% of *Paenibacillus* strains.

**Figure 4:**
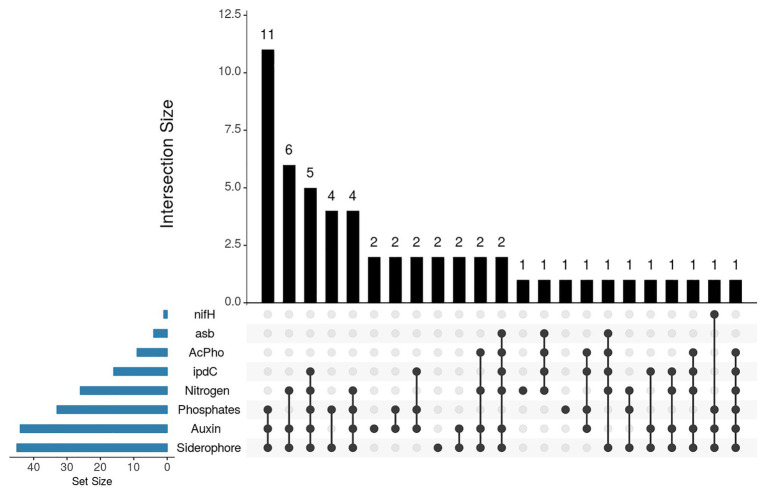
UpSet plot of PGP traits detected by biochemical assays and by PCR. The bar chart on the left indicates the total number of isolates that exhibit each PGP trait. The upper bar chart indicates the intersection size between sets of isolates with one or more PGP traits. Dark connected dots indicate which PGP trait is considered for each intersection.

Additionally, the frequency of strains with one or more PGP traits tested by both phenotypic and genotypic approaches was analyzed (Figure 4). The results showed that 11 strains constituted the largest functional group. They showed the phenotypic potential for phosphates solubilization, IAA synthesis and siderophore production, followed by six strains with phenotypic potential for nitrogen fixation, IAA, and siderophore production, and five isolates with phenotypic potential for all PGP traits and genotypic potential for IAA production. Interestingly, two isolates exhibited genotypic and phenotypic potential for tree PGP traits.

### Improved Germination in Cape Gooseberry Seeds

**Figure 5:**
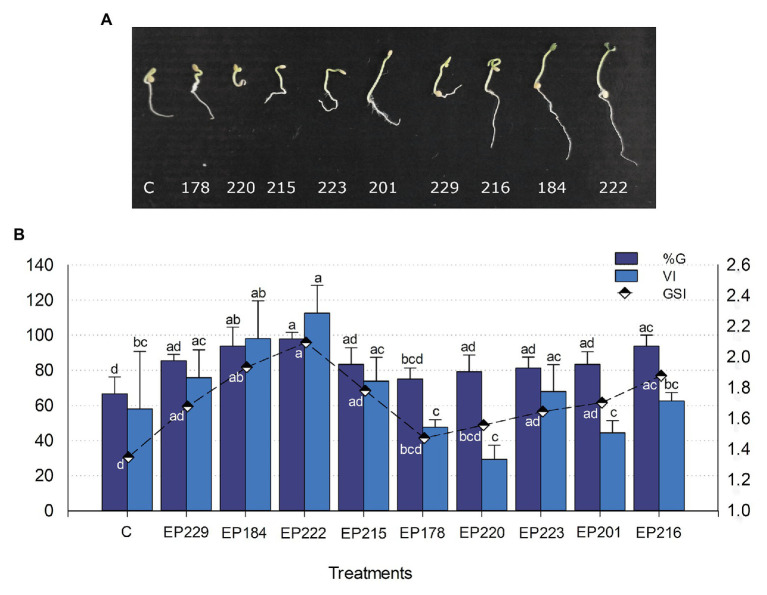
Effect of bacterial strains isolated from *P. incarnata* on the Cape gooseberry germination: **(A)** Representative pictures on sprouted seeds at 10 days and **(B)** Bar chart of germination percentage (%G) and vigor index (VI) exhibited by each bacterial strain. The vigor index (VI) is graphed with black and white colored rhombuses. Values represent the arithmetic mean ± SD. Treatments with different letters, within each tested parameter, are significantly different according to the Tukey statistical test (*p* < 0.05).

Based on results from biochemical PGP tests, nine endophytic strains were selected for testing their effect on germination percentage and speed and vigor index in Cape gooseberry seeds. All isolates increased the germination percentage by 6–29%, compared with the control. The EP222 (associated with *Pantoea ananatis*; 97.9%), EP184 (*B. megaterium*), and EP216 (*Leclercia adecarboxylata*; 93.8%) strains produced the highest germination percentages and were significantly different (*p* < 0.05) from non-inoculated seeds (66.7%). These strains reached the mentioned above germination percentages in just 10 days, and they also exhibited the highest GSIs (Figure 5A). The vigor index was increased in 2.7–52.7% by the strains EP222, EP184, EP229, EP215, EP223, and EP216 in comparison with the control treatment (Figure 5B). The strains EP222, EP184, and EP216 significantly increased germination parameters compared with control, which suggested that it could be used as an effective inoculant in Cape gooseberry seeds.

### Plant Growth Promotion in Cape Gooseberry Plants

**Figure 6:**
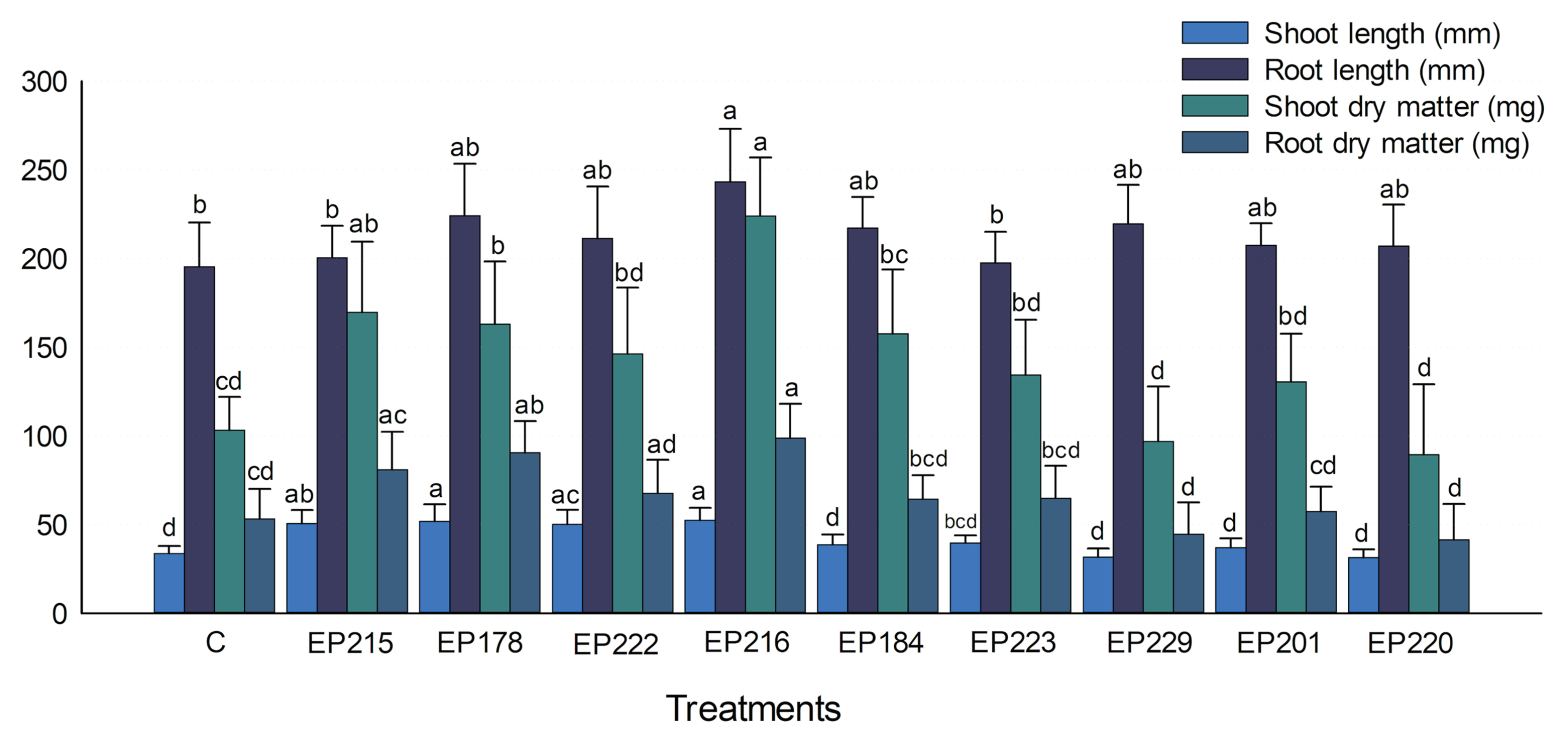
Effect of bacterial inoculants isolated from *P. incarnata* on the Cape gooseberry growth. Values represent the arithmetic mean ± SD. Treatments with different letters, within each tested parameter, are significantly different according to the Tukey statistical test (*p* < 0.05).

The results showed that the treatment with selected strains boosted the Cape gooseberry growth (Figure 6). Inoculation with strain EP216 exhibited the best results about the shoot and root lengths, increasing it by 55.4 and 24.5% compared with the control. Concerning to shoot and root dry matter, treatment with strain EP216 significantly improved these parameters compared with the control treatment, followed by strains EP215 and EP178. The individuals treated with strain EP184 also increased the shoot and root dry matter by 52.7 and 24.5%, respectively. All treatments showed increased phenotypic parameters in comparison with control, except the strains EP229 and EP220. The a and b chlorophyll levels were significantly higher in plants inoculated with EP216 than in control (Supplementary Table S3). The plants treated with strains EP184, EP223, and EP229 also showed higher chlorophyll levels than the control treatment. For the nutritional parameters measured in plant aboveground parts, the inoculation of isolates EP178, EP216, EP229, EP220, and EP201 showed nitrogen, phosphorus, potassium, calcium, copper, iron, manganese, and sodium levels higher than the control.

## Discussion

Plants can recruit beneficial microbes from the environment as response to a particular unfavorable condition (Liu et al., 2020). Nutritional stress that passionflowers face when growing in poor soils and/or without application of fertilizer can promote recruitment and accumulation of microorganisms with the capacity to cope with nutritional needs. Also, the occurrence of microorganisms with the potential to facilitate the acquisition of essential nutrients or modulate the level of hormones within plants might be substantial in the early growth stages. This study revealed that most endophytic bacteria retrieved in the vegetative stage of passionflower possess multiple PGP traits. The phenotypic and genotypic approaches were carried out to access these functional traits (Raddadi et al., 2008). Combining these approaches allowed us to confirm the potential attributed by one, complement the result between both, or increase detection coverage when ones failed to reveal the PGP trait.

The phylogenetic analysis of 16S rRNA gene sequences allowed taxonomically categorize endophytic isolates and revealed bacterial diversity of genera. In the case of the EP178 and EP223 strains, a further phylogeny analysis suggested that they may belong to a new species of *Pseudomonas*. Phylogenetic affiliations for *Bacillus* isolates were difficult because the 16S rRNA gene has low phylogenetic resolution and weak discriminatory power for some taxonomic groups (Ash et al., 1991; Janda and Abbott, 2017), so the use of another taxonomic marker is recommended. Endophytic isolates were mainly associated with *Bacillus* and *Pseudomonas*. The dominance of these genera was already reported in other medicinal plants (Miller et al., 2012; Rhoden et al., 2015). Coincidentally, members of *Bacillus* and *Pseudomonas* have been extensively reported as plant growth enhancers (McSpadden Gardener, 2004; Mercado-Blanco and Bakker, 2007; Govindasamy et al., 2010; Ferreira et al., 2019), suggesting what could be the ecological role of these dominant groups in the vegetative stage of *P. incarnata*. The next more abundant genus was *Pantoea*, which has already been reported in medicinal plants such as *Hypericum perforatum* and *Ziziphora capitate* (Egamberdieva et al., 2017); even this genus was the most found in six *Eucallyptus* species (Procópio et al., 2009). Although usually known as a plant pathogen, some studies reported *Pantoea* strains with plant growth-promoting capabilities (Andreolli et al., 2016; Chen et al., 2017). Likewise, several members of the less represented genera (*Lysinibacillus*, *Microvirga*, *Xanthomonas*, and *Leclercia*) in this study have been described previously as both endophytes, and plant growth promoters (Shahzad et al., 2017; Walitang et al., 2017; Shabanamol et al., 2018), reinforcing the hypothesis that bacterial endophytes naturally occurring in the vegetative stage of *P. incarnata* may have a crucial role in vegetative development of plant host.

Microbes often use two mechanisms to support the plant growth directly (Santoyo et al., 2016): (1) facilitating the mobilization and uptake of soil deficient nutrients (Van der Heijden et al., 2008) and (2) modulating the level of hormones in plants (Verbon and Liberman, 2016). In this study, the potential of isolates to mobilize essential nutrients (N, P, and Fe) and synthesize phytohormones (auxins) was accessed. Most strains reported in this study exhibited PGP traits in biochemical and/or genetic assays. Nitrogen is present abundantly in the environment in its diatomic form (N_2_), limiting its absorption for plants. Many strains grown in the NFb medium, forming a sub-surface pellicle, and they were mostly associated with the *Bacillus* genus. A previous study reported endophytic *Bacillus* strains isolated from the *Lolium perenne* rhizosphere, which showed their diazotrophic activity (Castellano-Hinojosa et al., 2016). Although most studies described diazotrophic bacteria from rhizospheric environments, some investigations have reported phyllosphere bacteria associated with the nitrogen fixation (Pati and Chandra, 1993; Desgarennes et al., 2014). Amplification of the *nifH* gene was performed to confirm diazotrophic potential. However, *nifH* sequences were detected in just one strain (EP220). The NFb medium not only allows the retrieval of diazotrophs but also favors the growth of bacteria able to scavenge traces of different nitrogen sources from the atmosphere (Zuluaga et al., 2020).

Phosphorus is an essential nutrient for plant development and growth (Khan et al., 2010). The isolates associated to *Bacillus*, *Pseudomonas*, and *Pantoea* were the most frequently encountered in the phosphate solubilization phenotypic tests. Some studies have shown that the main mechanism of *Pantoea* and *Pseudomonas* to solubilize inorganic phosphate is the secretion of the gluconic acid (Castagno et al., 2011; Oteino et al., 2015). In comparison, *Bacillus* species secrete other organic acids, such as lactic, acetic, succinic, and propionic (Saeid et al., 2018). Taking into account that the solubilization of inorganic phosphate compounds occurs mainly through the secretion of organic acids, changes in the composition of the culture medium may alter the microbial metabolism and affect the rate of solubilization (Nautiyal, 1999), masking the ability of strains for phosphate solubilization. The gene encoding phosphatase enzyme was chosen as a genetic marker since it is involved in the solubilization of various organic phosphates. The *AcPho* gene was amplified using primers designed from *B. thuringiensis* sequences (Raddadi et al., 2008), which favored the detection in *Bacillus* strains and limited it for the less represented groups. *AcPho* sequences were not only detected in strains closely associated with *B. thuringiensis* but also in other taxa (*B. aryabhattai*, *B. tequilensis*, *B. megaterium*, *B. anthracis*, and *B. cereus*), showing its potential as phosphate solubilization functional marker for the *Bacillus* genus. Overall, the combination of the two approaches proved to be complementary, since the formation of solubilizing halo on the NBRIP medium indicated that the bacteria could solubilize inorganic phosphate (Nautiyal, 1999). Meanwhile, the detection of *AcPho* sequences showed the potential to solubilize organic phosphates (Sharma et al., 2013). Various isolates confirmed their ability to solubilize organic and inorganic phosphate compounds, remarkably increasing their potential as plant beneficial inoculants.

The IAA is involved in several processes of plant vegetative development (Spaepen and Vanderleyden, 2011). The phenotypic assay to detect IAA-like compound production was carried out under the same culture conditions for all isolates, without taking into account their wide physiological and taxonomic diversity, which reduced the possibility of offering the specific conditions that each isolate requires to produce maximum IAA amounts (Frankenberger and Arshad, 1995). *Bacillus* was not only the genus with the most IAA-producing number of strains but also comprised the strain (EP229) with the highest value of IAA-like compounds. IAA values similar to this study were found in *Bacillus* isolates from plants at a multi-metal contaminated mine site (Shim et al., 2015). The gene (*ipdC*) chosen for the genotypic approach encodes a key enzyme in the main IAA biosynthetic pathway (IPA) found in plant-associated beneficial bacteria (Spaepen and Vanderleyden, 2011). The primers used to detect the ipdC gene were also designed on sequences belonging to *B. thuringiensis*, which favored its detection in *Bacillus* strains. Lyngwi et al. (2016) have already reported *ipdC* gene sequences in *Bacillus* and *Paenibacillus* species recovered from soils of the sacred groves in India. The *ipdC* sequences were also amplified in isolates belonging to *Rhodococcus*. A study previously demonstrated the ability of some *Rhodococcus* strains to synthesize IAA (Vandeputte et al., 2005). The isolates that produce IAA-like substances and harbor *ipdC* gene sequences could synthesize the IAA through the IPA pathway. However, the microbial IAA can be produced by other metabolic pathways, such as AMI or IAOx/IAN, which can occur and be expressed together with the IPA pathway (Duca et al., 2014).

Iron is the fourth most abundant element in the earth’s crust, but in aerobic (oxidant) conditions and neutral pH, it is almost insoluble for plants (Schwab and Lindsay, 1983). Under conditions of iron stress, microorganisms can produce low-molecular-mass compounds with high affinity for ferric ion, termed siderophores. Most of the tested strains secreted Fe (III) chelating agents when sequestered in the complex HDTMA-Fe (III)-CAS on the medium Blue Agar. This PGP trait was the most common among all identified genera as the method developed by Schwyn and Neilands (1987) did no limit the detection of a single molecule. Only *Lysinibacillus* and *Paenibacillus* strains did not exhibit siderophores-producing ability. Various studies previously reported siderophore production in *Bacillus*, *Pseudomonas*, and *Pantoea* strains (Loaces et al., 2011; Andreolli et al., 2016; Tchakounté et al., 2018). The *asb* gene was used as a genetic marker to characterize siderophore production because it commonly occurs in *Bacillus* species (Koppisch et al., 2008). However, this was amplified in just four *Bacillus* strains, likely because there is a wide structural diversity of siderophores described (Hider and Kong, 2010). On the other hand, a non-specific fragment related to the TatA/TatE subunit of the translocase A protein was amplified in several strains. Curiously, this protein is involved in the mechanism of the reception and translocation of an iron (III) reductase (Lechowicz and Krawczyk-Balska, 2015), so it is closely related to extracellular iron metabolism.

The treatment with the selected bacteria increased the germination rate of Cape gooseberry seeds, which often range 85–90% after 15 days of incubation (Fischer et al., 2005). The use of film-coating has already shown its potential to increase the germination rate of *P. peruviana* until 97% (Campos et al., 2015). However, this strategy must be carefully used as it may compromise the water and gas availability. In our study, the immersion of Cape gooseberry seeds in bacterial suspensions improved the germination rate and timing in 10 days. Some studies have reported PGP bacteria to positively influence seed germination synthesizing phytohormones (Delshadi et al., 2017). The bacteria used in the germination test showed potential for the synthesis of one of the main phytohormones (indole acetic acid) associated with vegetative development. The results from pot experiments supported the capability of the *P. incarnata* endophytic strains to promote plant growth. They have previously shown their potential to solubilize phosphates, synthesize IAA, and produce siderophores in genetic and biochemical assays. But, the detection of PGP traits by assays *in vitro* is not conclusive to determinate the effect of a candidate strain on the plant growth promotion, since that bacterial performance depends on environmental conditions and plant-microbe interactions (Smyth et al., 2011). However, selected strains were able to improve agronomic parameters in Cape gooseberry seedlings, suggesting that they might have used mechanisms exhibited *in vitro* to stimulate plant development and growth *in vivo*. The used endophytic strains as probiotic and protective agents for crops have gained relevance as they possess traits associated to improvement and supporting of plant development and health. In addition, they intrinsically have the capability of access to a restricted environment, the endosphere. The boosting effects of selected strains were exhibited in a plant species different than native, suggesting their versatility for colonizing other environments. These characteristics become them in promising candidates for agriculture systems (Farrar et al., 2014).

The function and structure of plant-associated microbiomes are shaped by host and environmental factors (Trivedi et al., 2020). However, a theory denominated “Cry for Help” hypothesize that a plant can attract beneficial microbes from the environment to cope with particular stresses (Liu and Brettell, 2019). This recruitment might also be associated to physiological needs that a plant exhibits during its development. The uptake of essential nutrients (such as N, P, K, and S) and the auxin synthesis are substantially higher in the early stages of plant development (Ramanathan and Krishnamoorthy, 1973; Ljung et al., 2001; Arunachalam and Chavan, 2018). Thus, the recruitment of bacteria capable meet these physiological needs (Carvalhais et al., 2013) may be favored during the passionflower vegetative development. Vendan et al. (2010) also found endophytic PGP bacteria mainly in the early stages of the ginseng life cycle. A study reported the dominance of IAA-producing rhizobacteria in the rosette (early) canola development stage (Farina et al., 2012). On the other hand, various studies have described PGP activities in microorganisms isolated from medicinal plants (Ansary et al., 2018; Li et al., 2018; Huang et al., 2019; Aeron et al., 2020). As in the food industry, pharmacological companies require that medicinal plant culture not includes chemical fertilization. This requirement reflects challenging conditions that plant medicinal culture deals and suggests a possible role of PGP microorganisms in these plants. The physiological needs that passionflower often exhibits in its vegetative stage added to the limited nutritional conditions that this plant faces in the agriculture systems of pharmacological interest which might explain the occurrence of PGP bacteria. However, a study of taxonomic and functional profile of endophytic microbiome could describe more precisely the bacterial group’s domaining endophytic environment and reveal roles they playing in the development and growth of plant host.

## Conclusion

This study reveals the dominance of groups belonging to *Bacillus*, *Pseudomonas*, and *Pantoea* among the bacterial endophytes from passionflower leaves. The contribution of these genera to the promotion of plant-growth and germination is highlighted by their potential to produce IAA, solubilize phosphate, and synthesize siderophores as demonstrated by the present assays using Cape gooseberry as a model. It can be also concluded that the combination of genotypic and phenotypic approaches is effective in revealing plant growth-promoting traits. The occurrence of several culturable PGP strains is probably associated with conditions of the passionflower culture. The strains of bacteria isolated in this study may be used in future projects as beneficial inoculants for agricultural systems.

## Data Availability Statement

The datasets presented in this study can be found in online repositories. The names of the repository/repositories and accession number(s) can be found below: Isolate sequences were deposited in GenBank under accession numbers MG778707 to MG778907.

## Author Contributions

LC-Y and FF-G designed the work and drafted the manuscript. LC-Y and MN conducted experiments *in vitro* and *in vivo*, respectively. MG, DA, and FF-G analyzed and interpreted the results. All authors read and approved the manuscript.

### Conflict of Interest

The authors declare that the research was conducted in the absence of any commercial or financial relationships that could be construed as a potential conflict of interest.

## Footnotes

^1^https://www.ezbiocloud.net/

^2^http://itol.embl.de
