# Supplementary material for: Multiple Plant Growth-Promotion Traits in Endophytic Bacteria Retrieved in the Vegetative Stage From Passionflower
Source: Front Plant Sci. 2021 Jan 18;11:621740. doi: 10.3389/fpls.2020.621740 (PMC7847900; doi:10.3389/fpls.2020.621740)
Supplement: Supplementary file 1 [file Table_1.docx]

***Supplementary Material***

**Supplementary Table S1**. Molecular identification of 58 bacterial isolates using 16S rDNA sequence analysis against EzBioCloud databases.

| Isolate ID | Closest neighbor | Sequence similarity (%) | GenBank ID |
| --- | --- | --- | --- |
| EP176 | *Paenibacillus barcinonensis* BP-23 | 99.10 | AJ716019 |
| EP177 | *Bacillus aryabhattai* B8W22 | 100 | EF114313 |
| EP178 | *Pseudomonas* sp. 1766 | 100 | SOBP01000022 |
| EP179 | *Bacillus tequilensis* KCTC 13622 | 100 | AYTO01000043 |
| EP180 | *Bacillus tequilensis* KCTC 13622 | 100 | AYTO01000043 |
| EP181 | *Bacillus aryabhattai* B8W22 | 100 | EF114313 |
| EP182 | *Bacillus tequilensis* KCTC 13622 | 100 | AYTO01000043 |
| EP183 | *Bacillus tequilensis* KCTC 13622 | 100 | AYTO01000043 |
| EP184 | *Bacillus megaterium* NBRC 15308 | 100 | JJMH01000057 |
| EP185 | *Bacillus paranthracis* Mn5 | 100 | MACE01000012 |
| EP186 | *Bacillus megaterium* NBRC 15308 | 100 | JJMH01000057 |
| EP187 | *Bacillus velezensis* CR-502 | 99.93 | AY603658 |
| EP188 | *Bacillus aryabhattai* B8W22 | 100 | EF114313 |
| EP189 | *Bacillus aryabhattai* B8W22 | 100 | EF114313 |
| EP190 | *Bacillus aryabhattai* B8W22 | 100 | EF114313 |
| EP191 | *Bacillus megaterium* NBRC 15308 | 100 | JJMH01000057 |
| EP192 | *Bacillus aryabhattai* B8W22 | 100 | EF114313 |
| EP193 | *Bacillus megaterium* NBRC 15308 | 100 | JJMH01000057 |
| EP194 | *Bacillus siamensis* KCTC 13613 | 99.93 | AJVF01000043 |
| EP195 | *Bacillus siamensis* KCTC 13613 | 99.92 | AJVF01000043 |
| EP196 | *Bacillus megaterium* NBRC 15308 | 100 | JJMH01000057 |
| EP197 | *Bacillus tequilensis* KCTC 13622 | 100 | AYTO01000043 |
| EP198 | *Bacillus altitudinis* 41KF2b | 100 | ASJC01000029 |
| EP199 | *Bacillus safensis* FO-36b | 100 | ASJD01000027 |
| EP200 | *Pantoea stewartii* LMG 263 | 99.77 | JPKO01000033 |
| EP201 | *Pseudomonas oryzihabitans* NBRC 102199 | 100 | BBIT01000012 |
| EP202 | *Bacillus wiedmannii* FSL W8-0169 | 100 | LOBC01000053 |
| EP203 | *Bacillus tequilensis* KCTC 13622 | 100 | AYTO01000043 |
| EP204 | *Pantoea vagans* LMG 24199 | 99.53 | EF688012 |
| EP205 | *Pantoea ananatis* LMG 2665 | 99.92 | JMJJ01000010 |
| EP206 | *Lysinibacillus fusiformis* NBRC 15717 | 99.92 | AB271743 |
| EP207 | *Xanthomonas sontii* PPL1 | 100 | NQYO01000058 |
| EP208 | *Rhodococcus erythropolis* NBRC 15567 | 99.92 | BCRM01000055 |
| EP209 | *Bacillus safensis* subsp. safensis FO-36b | 100 | ASJD01000027 |
| EP210 | *Bacillus megaterium* NBRC 15308 | 100 | JJMH01000057 |
| EP211 | *Bacillus megaterium* NBRC 15308 | 100 | JJMH01000057 |
| EP212 | *Paenibacillus xylanexedens* B22a | 99.46 | EU558281 |
| EP213 | *Bacillus megaterium* NBRC 15308 | 100 | JJMH01000057 |
| EP214 | *Bacillus mobilis* 0711P9-1 | 99.93 | MACF01000036 |
| EP215 | *Pseudomonas oryzihabitans* NBRC 102199 | 100 | BBIT01000012 |
| EP216 | *Leclercia* *adecarboxylata* NBRC 102595 | 99.6 | BCNP01000062 |
| EP217 | *Bacillus siamensis* KCTC 13613 | 99.84 | AJVF01000043 |
| EP218 | *Bacillus* *cereus* ATCC 14579 | 100 | AE016877 |
| EP220 | *Pseudomonas* *cichorii* ATCC 10857 | 99.12 | FNIK01000055 |
| EP221 | *Bacillus aryabhattai* B8W22 | 100 | EF114313 |
| EP222 | *Pantoea ananatis* LMG 2665 | 99.92 | JMJJ01000010 |
| EP223 | *Pseudomonas* sp. 1766 | 100 | SOBP01000022 |
| EP224 | *Bacillus megaterium* NBRC 15308 | 100 | JJMH01000057 |
| EP225 | *Rhodococcus* *erythropolis* NBRC 15567 | 100 | BCRM01000055 |
| EP226 | *Microvirga* *calopogonii* CCBAU 65841 | 99.92 | QDGA01000235 |
| EP227 | *Bacillus safensis* subsp. safensis FO-36b | 100 | ASJD01000027 |
| EP228 | *Bacillus aryabhattai* B8W22 | 100 | EF114313 |
| EP229 | *Bacillus megaterium* NBRC 15308 | 99.92 | JJMH01000057 |
| EP230 | *Bacillus safensis* subsp. safensis FO-36b | 100 | ASJD01000027 |
| EP231 | *Bacillus aryabhattai* B8W22 | 100 | EF114313 |
| EP233 | *Bacillus tequilensis* KCTC 13622 | 100 | AYTO01000043 |
| EP234 | *Bacillus taxi* M5HDSG1-1 | 99.36 | MK355518 |
| EP235 | *Bacillus gibsonii* DSM 8722 | 99.84 | X76446 |

**Supplementary Table S2**. Results from biochemical assays and amplification of PGP related genes.

| Strain ID | Nitrogen fixation | | Phosphate solubilization | | Siderophore production | | IAA production | |
| --- | --- | --- | --- | --- | --- | --- | --- | --- |
|  | NFb | *nifH* | SI | *AcPho* | CAS | *asb* | IAA(µg/ml) | *ipdC* |
| EP176 | - | - | 2.75 | - | - | - | 4.81 | + |
| EP177 | - | - | - | - | + | * | 3.74 | - |
| EP178 | - | - | 3.20 | - | + | - | 3.17 | - |
| EP179 | - | - | 2.50 | - | - | - | 3.27 | + |
| EP180 | - | - | 2.25 | + | - | - | 1.66 | + |
| EP181 | + | - | - | + | + | - | 4.02 | + |
| EP182 | + | - | - | - | + | - | 1.01 | - |
| EP183 | + | - | 2.54 | - | + | - | 1.28 | + |
| EP184 | + | - | 2.14 | - | + | * | 3.93 | - |
| EP185 | + | - | - | + | + | + | 1.32 | + |
| EP186 | + | - | - | - | + | * | 2.41 | - |
| EP187 | - | - | - | - | + | * | - | - |
| EP188 | + | - | 2.22 | - | + | - | 2.38 | + |
| EP189 | + | - | 2.23 | + | + | - | 1.35 | + |
| EP190 | + | - | - | + | + | * | 4.7 | - |
| EP191 | + | - | - | - | + | * | 3.9 | - |
| EP192 | + | - | 2.15 | - | + | - | 2.1 | - |
| EP193 | + | - | - | + | + | * | 1.33 | - |
| EP194 | - | - | - | - | + | * | - | - |
| EP195 | + | - | 2.22 | - | + | - | 3.81 | + |
| EP196 | - | - | 2.13 | - | + | * | 2.29 | - |
| EP197 | + | - | - | - | + | - | 1.17 | - |
| EP198 | - | - | 2.28 | - | + | - | - | - |
| EP199 | - | - | - | - |  | - | - | - |
| EP200 | - | - | 2.07 | - | + | - | 2.77 | - |
| EP201 | - | - | 3.33 | - | + | - | 3.1 | - |
| EP202 | + | - | - | + | + | + | 3.65 | + |
| EP203 | - | - | 2.33 | - | + | - | - | - |
| EP204 | - | - | 2.30 | - | + | - | 3.39 | - |
| EP205 | - | - | 2.22 | - | + | - | 2.07 | - |
| EP206 | - | - | - | - | - | - | - | - |
| EP207 | - | - | 2.65 | - | + | - | 1.28 | - |
| EP208 | + | - | - | - | - | - | - | - |
| EP209 | - | - | 2.25 | - | - | - | - | - |
| EP210 | - | - | 2.33 | - | + | - | 1.59 | - |
| EP211 | + | - | - | - | + | * | 2.19 | - |
| EP212 | - | - | 2.33 | - | - | * | 5.88 | - |
| EP213 | + | - | 2.10 | - | + | - | - | - |
| EP214 | + | - | - | + | - | + | - | + |
| EP215 | - | - | 3.48 | - | + | - | 5.14 | - |
| EP216 | - | - | 3.20 | - | + | - | 3.08 | - |
| EP217 | - | - | - | - | + | - | 3.07 | - |
| EP218 | + | - | - | + | + | + | - | + |
| EP220 | - | + | 3.00 | - | + | * | 5.3 | - |
| EP221 | + | - | 2.26 | - | + | * | 2.29 | + |
| EP222 | - | - | 2.70 | - | + | - | 3.32 | - |
| EP223 | - | - | 3.53 | - | + | - | 2.44 | - |
| EP224 | + | - | - | - | + | - | 1.91 | - |
| EP225 | + | - | 2.67 | - | + | - | 1.89 | + |
| EP226 | - | - | 2.53 | - | + | - | - | - |
| EP227 | - | - | - | - | - | - | - | - |
| EP228 | + | - | 2.27 | - | + | * | 2.05 | - |
| EP229 | + | - | 2.33 | - | + | * | 6.04 | - |
| EP230 | - | - | - | - | - | - | - | - |
| EP231 | + | - | - | - | + | - | 5.44 | + |
| EP233 | - | - | - | - | + | - | 1.7 | + |
| EP234 | - | - | 2.40 | - | - | - | 4.18 | - |
| EP235 | - | - | - | - | - | - | 2.99 | - |

* = unspecific and partial amplification of the *tatA* gene.

The numerical values for tested traits are means (n=3)

**Supplementary Table S3.** Effect of bacterial inoculants isolated from *Passiflora incarnata* on production of A and B chlorophyll and accumulation of macro- and micronutrients in the Cape gooseberry aboveground parts.

| Inoculation treatment | Chlorophyll* | | Macronutrients | | | | Micronutrients | | | |
| --- | --- | --- | --- | --- | --- | --- | --- | --- | --- | --- |
|  | A | B | N | P | K | Ca | Fe | Cu | Mn | Na |
| Control | 31.0 ± 1.6**bc** | 6.6 ± 1.0**bc** | 24.1 ± 1.4 | 3.38 ± 0.37 | 49.1 ± 2.8**ab** | 4.7 ± 0.3**b** | 179.93 ± 7.1 | 6.0 ± 0.1**ab** | 92.4 ± 9.1**ab** | 4065.2 ± 756.2**ab** |
| EP215 | 30.9 ± 1.8**bc** | 6.4 ± 0.9**c** | 19.9 ± 1.5 | 3.03 ± 0.26 | 40.8 ± 3.3**b** | 4.7 ± 0.3**a** | 215.83 ± 37.5 | 5.3 ± 0.5**b** | 86.5 ± 0.1**b** | 2929.9 ± 580.4**ab** |
| EP178 | 30.2 ± 1.8**c** | 6.2 ± 0.8**c** | 24.5 ± 1.4 | 3.83 ± 0.48 | 52.2 ± 6.1**a** | 6.2 ± 0.6**b** | 186.50 ± 29.6 | 7.5 ± 0.6**a** | 124.9 ± 20.9**a** | 2596.5 ± 1057.4**ab** |
| EP222 | 30.4 ± 1.8**c** | 6.4 ± 0.7**c** | 24.3 ± 5.6 | 3.51 ± 0.30 | 48.7 ± 2.3**ab** | 4.9 ± 0.4**ab** | 226.67 ± 79.3 | 6.3 ± 0.5**ab** | 96.7 ± 16.8**ab** | 3533.3 ± 1331.7**ab** |
| EP216 | 34.5 ± 1.6**a** | 8.2 ± 1.0**a** | 25.5 ± 2.2 | 2.97 ± 0.25 | 42.8 ± 3.4**ab** | 4.9 ± 0.4**ab** | 158.03 ± 12.3 | 5.2 ± 0.4**b** | 71.2 ± 3.6**b** | 2450.3 ± 798.2**b** |
| EP184 | 33.5 ± 1.0**ab** | 8.0 ± 0.8**ab** | 25.3 ± 5.6 | 3.32 ± 0.15 | 51.5 ± 4.2**a** | 4.9 ± 0.7**b** | 181.20 ± 4.5 | 5.5 ± 0.3**b** | 62.5 ± 1.6**b** | 3247.0 ± 422.6**ab** |
| EP223 | 32.0 ± 1.9**abc** | 6.8 ± 0.7**ac** | 22.5 ± 3.7 | 3.19 ± 0.21 | 49.0 ± 4.2**ab** | 4.6 ± 0.2**b** | 175.27 ± 20.5 | 5.2 ± 0.1**b** | 93.1 ± 11.0**ab** | 2861.1 ± 120.6**ab** |
| EP229 | 32.0 ± 2.2**abc** | 6.9 ± 1.2**ac** | 26.6 ± 1.8 | 3.43 ± 0.20 | 44.7 ± 2.3**ab** | 4.5 ± 0.3**b** | 159.80 ± 24.3 | 6.2 ± 0.1**ab** | 100.0 ± 9.1**ab** | 3920.1 ± 302.0**ab** |
| EP201 | 30.6 ± 1.5**bc** | 6.3 ± 0.7**c** | 26.9 ± 2.8 | 3.56 ± 0.54 | 52.4 ± 3.4**a** | 5.0 ± 0.3**ab** | 164.50 ± 11.9 | 5.6 ± 0.6**b** | 82.7 ± 15.9**b** | 4063.0 ± 614.3**ab** |
| EP220 | 29.8 ± 2.2**c** | 6.1 ± 0.8**c** | 25.8 ± 2.1 | 3.49 ± 0.05 | 48.7 ± 2.9**ab** | 5.2 ± 0.6**ab** | 159.67 ± 27.2 | 5.6 ± 0.6**b** | 109.0 ± 8.0**ab** | 4533.3 ± 115.5**a** |

Data are expressed as mean ± SD. Values with different letters within the columns are significantly different according to the Tukey test (p < 0.05).

*Index of Falker chlorophyll. This a dimensionless index, proportional to gram of chlorophyll per gram of leaf.

*Pseudomonas stutzeri* ATCC 17588 (CP002881)

*Pseudomonas flexibilis* ATCC 29606 (JRUD01000023)

*Pseudomonas alcalophila* AL15-21 (AB030583.1)

*Pseudomonas chengduensis* MBR (EU307111)

*Pseudomonas toyotomiensis* HT-3 (AB453701)

***Pseudomonas* sp. EP 178; *Pseudomonas* sp. EP 223**

*Pseudomonas oleovorans* IAM 1508 (D84018.1)

*Pseudomonas oryzihabitans* NBRC 102199 (BBIT01000012)

*Pseudomonas psychrotolerans* C36 (AJ575816.1)

*Azorhizophilus paspali strain* NBRC 102228 (NR 114054.1)

**98**

**69**

**100**

**80**

**100**

**49**

**39**

0.005

# Supplementary Figure S1. Neighbohr-Joining phylogenetic tree of *Pseudomonas* species based on the partial 16S rRNA gene sequences of EP178 (MG778852) and EP223 (MG778896) strains and type material. The analysis was based on 1405 positions. The scale bar represents an estimated 5 base substitutions per 1000 nt positions. Percentages refer to significant bootstrap values of 1000 calculated trees. *Azorhizophilus paspali* NBRC 102228 was used as outgroup.

**Supplementary Figure S2**. Classification bacterial genera with capacity to solubilize inorganic phosphate according with Silva Filho and Vidor (2000).


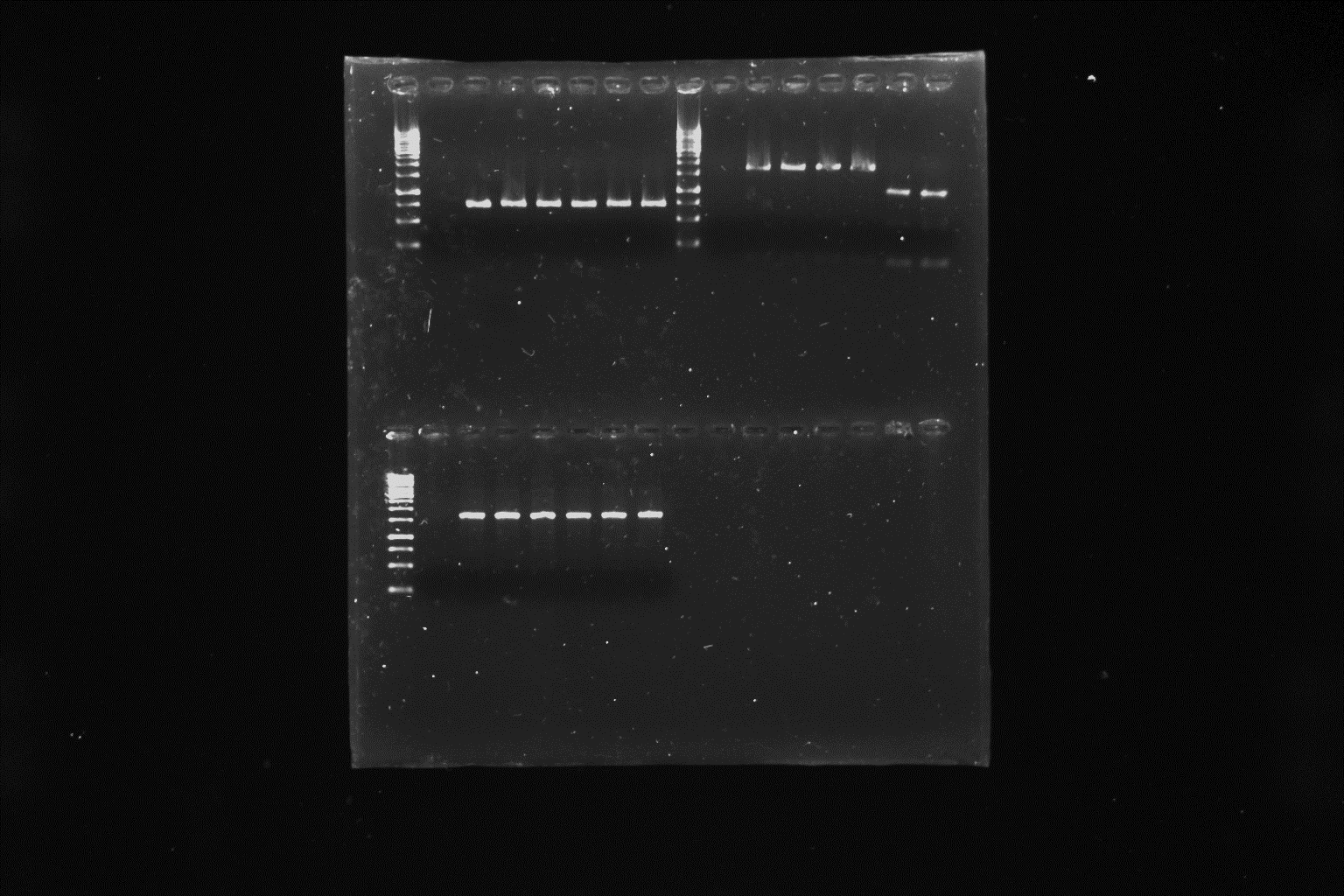


**1500**

**1000**

**750**

**500**

**250**

**2000**

**PM**

**C-**

**185**

**202**

**214**

**218**

**190**

**193**

**1685 bp**

**~1000 bp**

**Supplementary Figure S3**. Amplification of the *asb* gene in the EP185, EP202, EP214, and EP218 isolates. Nonspecific fragments for the EP190 and EP193 isolates. C -: negative control.
